# Magnetic resonance imaging for adaptive cobalt tomotherapy: A proposal

**DOI:** 10.4103/0971-6203.29194

**Published:** 2006

**Authors:** Tomas Kron, David Eyles, L John Schreiner, Jerry Battista

**Affiliations:** Peter MacCallum Cancer Centre, Melbourne, Australia; * Cancer Centre of Southeastern Ontario, Kingston Ontario, Canada; ** London Regional Cancer Program, London Health Sciences Centre and Department of Medical Biophysics, University of Western Ontario, Ontario, Canada

**Keywords:** Image guided radiation therapy, magnetic resonance imaging, tomotherapy

## Abstract

Magnetic resonance imaging (MRI) provides excellent soft tissue contrast for oncology applications. We propose to combine a MRI scanner with a helical tomotherapy (HT) system to enable daily target imaging for improved conformal radiation dose delivery to a patient. HT uses an intensity-modulated fan-beam that revolves around a patient, while the patient slowly advances through the plane of rotation, yielding a helical beam trajectory. Since the use of a linear accelerator to produce radiation may be incompatible with the pulsed radiofrequency and the high and pulsed magnetic fields required for MRI, it is proposed that a radioactive Cobalt-60 (^60^Co) source be used instead to provide the radiation. An open low field (0.25 T) MRI system is proposed where the tomotherapy ring gantry is located between two sets of Helmholtz coils that can generate a sufficiently homogenous main magnetic field.

It is shown that the two major challenges with the design, namely acceptable radiation dose rate (and therefore treatment duration) and moving parts in strong magnetic field, can be addressed. The high dose rate desired for helical tomotherapy delivery can be achieved using two radiation sources of 220TBq (6000Ci) each on a ring gantry with a source to axis-of-rotation distance of 75 cm. In addition to this, a dual row multi-leaf collimator (MLC) system with 15 mm leaf width at isocentre and relatively large fan beam widths between 15 and 30 mm per row shall be employed. In this configuration, the unit would be well-suited for most pelvic radiotherapy applications where the soft tissue contrast of MRI will be particularly beneficial. Non-magnetic MRI compatible materials must be used for the rotating gantry. Tungsten, which is non-magnetic, can be used for primary collimation of the fan-beam as well as for the MLC, which allows intensity modulated radiation delivery. We propose to employ a low magnetic Cobalt compound, sycoporite (CoS) for the Cobalt source material itself.

Rotational delivery is less susceptible to problems related to the use of a low energy megavoltage photon source while the helical delivery reduces the negative impact of the relatively large penumbra inherent in the use of Cobalt sources for radiotherapy. On the other hand, the use of a ^60^Co source ensures constant dose rate with gantry rotation and makes dose calculation in a magnetic field as easy as the range of secondary electrons is limited.

The MR-integrated Cobalt tomotherapy unit, dubbed ‘MiCoTo,’ uses two independent physical principles for image acquisition and treatment delivery. It would offer excellent target definition and will allow following target motion during treatment using fast imaging techniques thus providing the best possible input for adaptive radiotherapy. As an additional bonus, quality assurance of the radiation delivery can be performed *in situ* using radiation sensitive gels imaged by MRI.

The ultimate goal of radiotherapy is to deliver a high radiation dose to a target while minimising the dose to surrounding healthy tissues. This requires both the ability to delivery highly conformal dose distributions as well as the localisation of the target every time radiation is delivered. While particle accelerators may in principle provide the best dose distributions,[1–3] intensity modulated radiation therapy (IMRT) with photons achieves very good dose delivery in practice[4–6] since the precise selection of intensity modulated photon beams can provide excellent conformal dose delivery. IMRT has advanced consider-ably, in particular through the development of multi-leaf collimators (MLCs), computer control of linear accelerators (linacs) and computerised inverse treatment planning in which beam parameters are calculated using optimisation algorithms and constraint criteria.[7]

It is now common to use the information from multiple imaging modalities for target definition during treatment planning.[8–10] Both magnetic resonance imaging (MRI)[11,12] and positron emission tomography (PET)[13–15] provide essential information, which improves target outlining in many clinical scenarios. However, improved target definition is not only required in treatment planning but also during treatment delivery where the position of the target can vary from day to day.[15–18] It would be difficult to perform daily PET scans with radioactive tracers for every treatment fraction, however, there is scope to utilise a high quality imaging tool during patient set-up on the treatment unit. This is usually referred to as image guided radiotherapy (IGRT) and the aim of the present paper is to develop a design for MRI based IGRT.

## Image-guided radiotherapy

Since the dose distributions resulting from 3D conformal therapy are specifically designed to fit tightly about the target volumes, there is an increased possibility of missing the target due to patient set-up errors organ motion or even small fluctuations in treatment delivery. Considerable work is underway to develop corrective image guided radiation therapy techniques to enable patient set up or radiation delivery, to be modified throughout a patient's treatment course using systematic feedback of various imaging and, perhaps, dose measurements made immediately prior to or during, treatment.[19–23]

One of the first imaging modalities included in the treatment room has been ultrasound. The NOMOS BAT system was designed to localise the prostate with the patient in treatment position and establish a correlation between the gland and the co-ordinate system of the radiotherapy treatment unit.[24] After appropriate operator training the system has proven to be useful for prostate patient positioning in a number of external beam radiotherapy procedures including IMRT.[25–26] A recent improvement has been the introduction of three dimensional ultrasound which is also available from another manufacturer.[27] However, there has been some discussion about the reproducibility of the ultrasound procedure and particularly the fact that the very ultrasound measurement applies pressure onto the abdomen thereby moving the prostate.[28–29] In addition to this, the ultrasound image does not allow to identify the external contour of the patient, thereby making it impossible to reconstruct the delivered dose. Dose reconstruction has been seen as the ultimate treatment verification as it determines the dose as it was delivered during treatment from data acquired during treatment.[30–34]

Therefore, a number of groups are investigating CT imaging as part of the daily treatment process. There are a variety of different approaches ranging from megavoltage (MV) fan beams in helical tomotherapy[35,36] to MV cone beam CT[37] and on board kV imaging devices with cone beam CT capability for on-line image guidance.[38–40] Others have approached improved image guidance by adding CT scanners into the treatment room.[41,42] Many of these systems are clinically in use for a few years and CT image guidance has proved useful for a number of clinical indications.[6,43–46]

However, while cone beam CT may enhance the ability to locate the patient from bony anatomy directly during treatment, additional imaging may be required in select sites to unambiguously define targets and organs at risk.[47–50] To this end it may be advantageous to incorporate MR imaging on a radiation treatment unit: MRI in this context replaces ultrasound or cone beam CT as integral part of a treatment unit; however, MRI is significantly more versatile and offers superior soft tissue contrast.

### Helical tomotherapy

**Figure 1:**
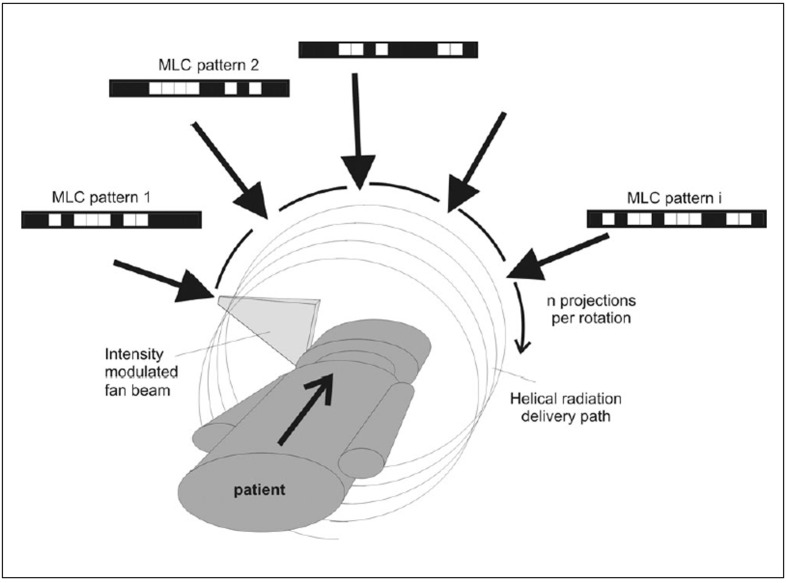
Illustration of the helical tomotherapy delivery

Recently, helical tomotherapy (HT) has been introduced as an IMRT device where a megavoltage linear accelerator (linac) continuously revolves around a patient, while slowly advancing the patient through the plane of rotation.[4,51,52] The concept, which is similar to helical computed tomography (CT), is illustrated in Figure 1. For radiation therapy dose delivery, a binary multileaf collimator (bMLC) is used to allow only sections of the fan beam to reach the patient that contribute to target irradiation in a desirable fashion. The bMLC consists of leafs which open and close very fast with transit times of the order of 20 m/s. Therefore, leaf positions can be considered to be binary, either open or shut with variable duration. The bMLC pattern changes as a function of gantry position, which provides many degrees of freedom to deliver highly conformal dose distributions. In practice, the rotational delivery is divided into n distinct projections as indicated in Figure 1. The commercial HT unit HiArt (TomoTherapy Inc., www.tomotherapy.com) employs 51 projections per rotation and each projection is characterised by a different leaf-opening pattern and duration profile.

### Magnetic resonance imaging

MRI is one of the most important imaging modalities in modern radiology. In addition to excellent soft tissue contrast, MRI can provide images based on flow or metabolic activity.[53] Consequently, MRI is increasingly used for radiotherapy treatment planning.[54–57] The soft tissue contrast which makes MRI useful for treatment planning especially for brain[58,59] and pelvic[50,60,61] lesions would clearly also be useful during the positioning of patients for daily treatment.

As such, it would be ideal to combine a MRI scanner with a radiotherapy treatment unit. However, it is very difficult to combine a medical linear accelerator with an MR unit as recently proposed by Raaymakers *et al.*[62] with the major problem being the need to de-couple the linear accelerator from all magnetic fields of the MRI scanner (static, gradient and RF) and vice versa.

It is the aim of the present paper to propose and describe a treatment unit that combines a helical tomotherapy unit with a MRI scanner. This is not meant to replace CT based IGRT units but to complement them in clinical scenarios where soft tissue contrast is essential. To avoid interference of magnetic fields between MR unit and linear accelerator, we propose to use of radioactive ^60^Co instead of X-rays produced by an accelerator as the radiation source. A similar approach has been taken by a group in Florida, US, which has developed a Cobalt-based treatment unit incorporated in an MR scanner (‘Renaissance™’, http://www.viewray.com/). The viewray system is based on three Cobalt sources and conventional multi-leaf collimators – it also integrates a sophisticated motion compensation method and the company hopes to have such as system available in 2008. In contrast to the viewray system, the present proposal is centred around helical tomotherapy[51,52] as the delivery mode. After a brief overview of the proposed system a detailed description of its components is provided. Emphasis is given here to major design challenges, such as having moving parts in a strong magnetic field and achieving the dose rate required for IMRT.

## Design overview of a combined MRI Co-60 tomotherapy unit

**Figure 2:**
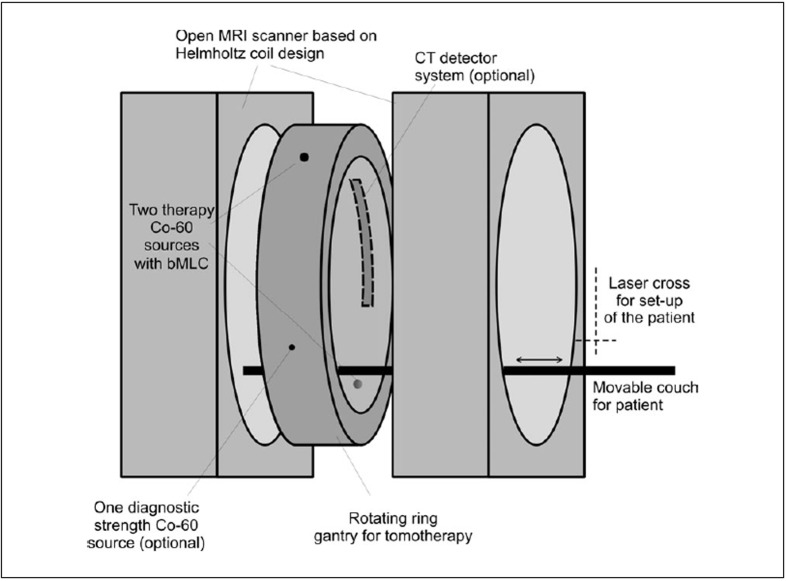
Schematic drawing of the Integrated MRI Cobalt tomotherapy unit

Figure 2 illustrates the basic layout of the proposed treatment unit. The main magnetic field of the MRI scanner is created by a set of Helmholtz coils. We propose that the magnetic field strength for the MRI Co tomotherapy unit should be of the order of approximately 0.25T, which allows conventional electromagnetic coils to be used.

Between the two sets of coils a ring gantry is to be mounted which accommodates two Co-60 sources. The patient will be protected by a thin stationary tunnel from the gantry, which rotates with an angular velocity variable between 10 and 120 sec. per rotation. As in the commercial HT unit, HiART (Tomotherapy Inc. Madison WI), the photon beam will be collimated to fan beam geometry. Intensity modulation will be achieved using a binary MLC with leaf motion parallel to the patient's movement through the gantry. The MLC opening pattern as a function of gantry angle determines the treatment delivery and must be optimised for individual patients using an inverse treatment planning process.[63]

**Figure 3:**
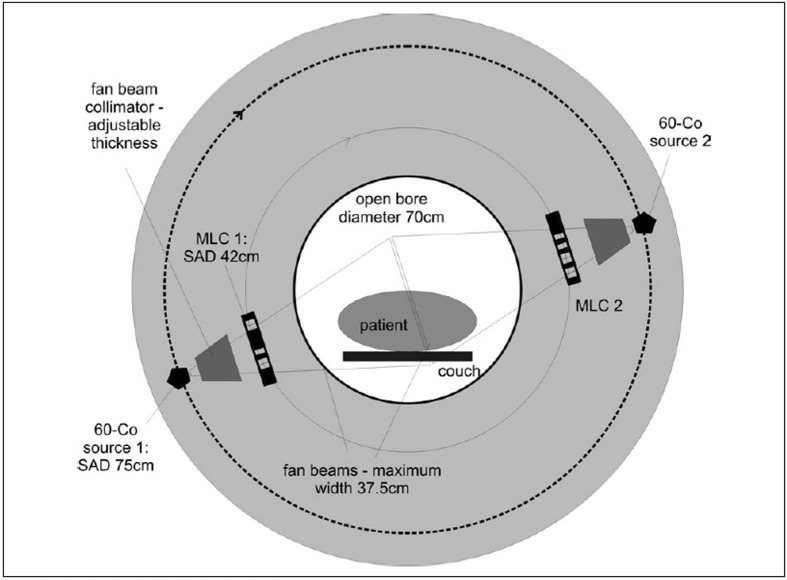
Frontal view of the proposed unit with dimensions

The patient is positioned on a flat carbon fibre couch, which moves through the both the rotating tomotherapy ring gantry and the two donuts of the Helmholtz MR coils. Couch speed will depend on the treatment scenario but would typically not exceed 5 cm per minute, which is slower than in most diagnostic helical CT protocols. As indicated in Figure 3, the tunnel for the patient is proposed to be 70 cm in diameter, which is comparable to most diagnostic CT scanners and somewhat larger than most MRI units. Due to the rotational intensity modulated beam delivery no special patient positioning devices such as breast or belly boards are required which will make a 70 cm bore diameter suitable for most patients and most treatment scenarios. Problems with claustrophobia will be similar to most MR units.

### Movement of objects in a strong magnetic field

One of the more significant problems in the design will be the continuous rotation of a rather large metal object within the magnetic fields required for the MRI data acquisition (if these are to be energised during treatment). In addition to this, the MLC leaves will move very quickly during opening and shutting. Fortunately, tungsten is non-magnetic and all other components can be made from other MR compatible materials. We propose to use sycoporite (CoS), a compound with high cobalt contents and low magnetic permeability for the radioactive Co 60 source. This is discussed in more detail below. The choice of relatively low field strength in the initial design for the main magnet allows one to choose from materials well tested in past and current diagnostic MR environments. In particular in interventional radiology and intraoperative imaging open MR scanners with relatively low field strength are quite common[64–67] and reduce safety concerns such as RF heating.[68,69]

If necessary, a simple way to overcome problems for the image acquisition in the presence of a rotating gantry would be to acquire MRI images just prior to and/or after the treatment while the gantry is stationary. As MR imaging does not constitute a direct hazard for the patient or staff, imaging can be performed as often as needed, for example prior and after the treatment delivery with the view to determine any motion, which occurred during treatment delivery. The MR scanner and its fields can be ‘shimmed’ for optimal image acquisition while the gantry is stationary and all magnetic fields could be deactivated after imaging before the gantry starts rotating. A similar ‘turn-off’ feature has been reported by Yrjana *et al.*[70] for neurosurgery applications. Turning off the magnet could also minimise power consumption and hazards associated with magnetic fields (e.g., magnetization of nearby devices and accessories).

In general, it becomes more common to combine the functionality of more than one modality in a single unit. Examples for this are PET/CT scanners[71,72] and most recently a hybrid MRI X-ray fluoroscopy system.[73] The latter combines a 0.5T open MRI system with a diagnostic X-ray fluoroscopy system. No significant deterioration in image quality within the magnet was observed which indicates that MRI can be combined with radiation emitting devices.

## Radiation delivery system

### Binary MLC

**Figure 4:**
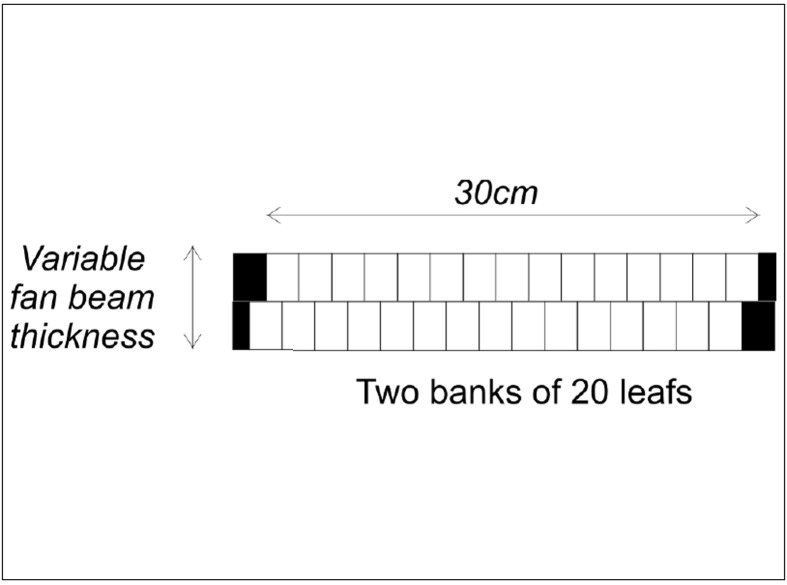
Beam's eye's view of the dual row multi-leaf collimator. The pair allows treatment of two adjacent slices of the patient during gantry rotation

The choice of two sources in the proposed design is a compromise between maximising dose rate and increasing complexity and cost associated with additional MLC systems and source replacements. The efficiency of dose delivery is also increased by using two adjacent rows of MLCs similar to the NOMOS MiMIC system.[74,75] The proposed MLC configuration is illustrated in Figure 4. It consists of two rows of 25 leafs, each 1.5 cm wide extending over a total fan beam width of 37.5 cm projected to the axis of rotation of the gantry. The fan beam thickness will be variable; however, for pelvic radiotherapy it is likely sufficient to incorporate only three options of 1.5, 2 and 3 cm per row. It is proposed to offset the two rows of leaves by half the leaf width against each other, to produce better spatial control of dose delivery within an axial plane of the patient. This also counteracts the effect of the relatively large width of individual leaves and adds additional capability for dose modulation in the planning process if a pitch factor (couch movement per gantry rotation in units of beam thickness) of less than 1 is used.

The widths of the individual MLC leaves are larger than those used in either the NOMOS MiMIC system (North American Scientific, Chatsworth, CA) or the HiART system of TomoTherapy Inc (Madison, WI). The dimensions were chosen to be compatible with the relatively large penumbra of Co 60 radiation beams[76] and the aims and typical margins for pelvic radiotherapy. The relatively large individual leaves reduce the complexity of the system by maximising the radiation fluence available to treat the target. With custom-designed sources, narrower companion collimation systems may be feasible in the future for other tumour sites that demand higher spatial control of the dose distribution, including penumbral regions.

Mackie *et al.*[51] have discussed the requirements for MLC leaf speed in their original publication introducing the helical tomotherapy concept. In the case of two sources with two MLC rows each, the requirements can be somewhat relaxed as the impact of each leaf motion only affects one of four beam delivery pathways. An acceptable transit time for the leafs of the MLC would be 30 m/s, which is similar to that achieved in the commercial HiArt system.[77]

As a note aside, the use of two rows in the MLC will also reduce the impact of the ramp up and down effect on the dose distribution in superior/inferior direction due to the helical dose delivery.[78] For example, with a pitch factor of 0.5, the first and last rotation of the gantry would only open leaves in one row of the MLC which reduces the dose due to a large fan beam thickness to areas inferiorly and superiorly of the target. If this technique is employed, the penumbra superiorly and inferiorly of the target becomes compatible or even smaller than in the commercial HT X-ray systems.

### Beam delivery and monitoring system

**Figure 5:**
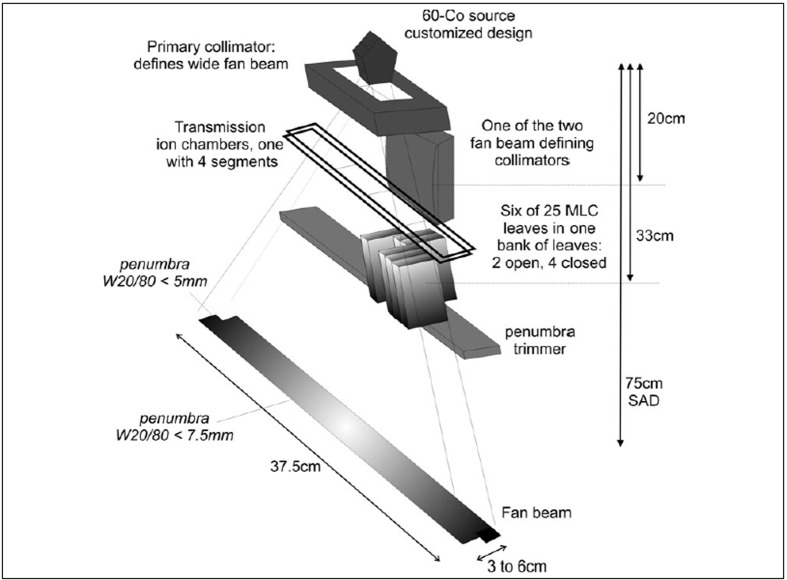
Illustration of all the components in the fan beam delivery system

Figure 5 illustrates the other components of the beam delivery system and indicates the dimensions considered to be most suitable. Most components in or close to the beam path are made from tungsten, since it has excellent radiation shielding characteristics and is a paramagnetic material with a low magnetic susceptibility (molar magnetic susceptibility = 5.3×10^−5^ cm^3^/mole). After a 10 cm thick primary collimator that attenuates the Co-60 photons by more than 10 half-value layers (HVL), the beam is further collimated to fan-beam geometry of adjustable width. The edges of the fan beam defining collimator can be slightly rounded to provide better definition of beam edges for a relatively large radiation source. This applies particularly to collimator sets close to the source where alignment of a flat collimator edge to an extended source is not possible.

Between the fan beam defining collimator and the MLC is a set of two transmission ionisation chambers. A rare but potentially serious problem with Cobalt 60 sources is the movement of radioactive pellets within the source capsule.[76] This may be more of concern in a strong magnetic field even when a low magnetic Cobalt compound is used. Therefore, only one of the transmission chambers is a single chamber that monitors the whole fan beam while the second is segmented into four adjacent segments that can monitor flatness and symmetry of the beam, which could be indicative of source dislocation.

Beyond the MLC, a 2 cm thick additional tungsten collimator is mounted parallel to the fan beam collimator. This collimator moves with the fan beam defining collimator and reduces the geometric penumbra of the beam in the patient's superior/inferior direction. It is important to note that the penumbra in superior/inferior direction consists of two components:

- If only one leaf in the two banks shown in Figure 4 is open, the penumbra at central axis it is defined by the MLC leaf edge of the closed collimator.
- The outer penumbra will be defined by the penumbra trimmer not the MLC leaf, which will always move further out of the beam than the maximum fan beam thickness. The pneumatic motion would not allow to adjust the leaf position accurately enough for different fan beam thicknesses.

As the fan beam thickness is typically fixed in any helical tomotherapy treatment neither the primary nor the secondary fan beam defining collimator would move during treatment which will simplify the drive mechanism.

### Cobalt sources

The tomotherapy delivery concept easily accommodates two or more radioactive sources on a single ring gantry. This contrasts significantly with the use of a linear accelerator as radiation source, in which the beam control hardware required for operation is complex and significant in size. The use of Co-60 has several additional advantages:

- It provides stable output without the need to provide power to an X-ray tube or linear accelerator via a slip ring.
- The output does not vary with gantry position.
- The beam delivery does not require beam steering hardware or high voltage components (which would likely add unwanted radiofrequency noise.
- The quality assurance and maintenance requirements of the radiation delivery components of the system is reduced (except for source changes every few years).

### Source activity, source size and treatment time

The specific activity of the sources, the target extent in superior/inferior direction and the degree of intensity modulation required determine the overall treatment time. In general, 10 minutes can be considered as an acceptable treatment time for a highly conformal intensity modulated radiotherapy procedure. This would be similar or even shorter than many conventional IMRT or tomotherapy delivery times.[79,80]

In general, the higher the source activity is, the shorter the treatment time will be. On the other hand, the source size must be kept as small as possible to reduce penumbra width and variations in output within the field due to partial source occlusion. Therefore, source and treatment head design must be a compromise between maximising dose rate and minimising effective size.

**Figure 6:**
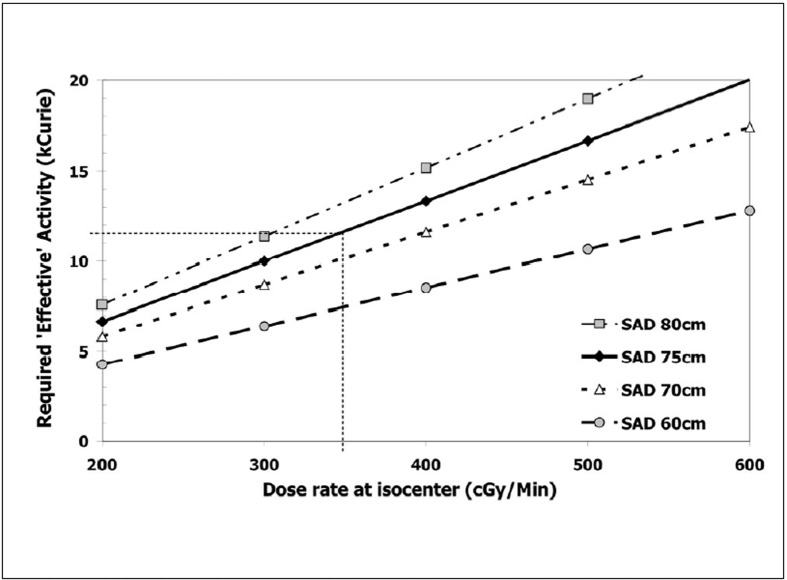
Effective activity required for a specified dose rate at the centre of gantry rotation. The activity was calculated for different source to axis distances and a single radioactive source

Figure 6 shows the required effective activity, A_eff,_ of a Cobalt-60 source on a ring gantry for a reference fan beam field size (FBFS) of 4×37.5 cm^2^. The calculation of A_eff_ for a required dose rate (RDR) at depth of maximum dose (d_max_) in the reference FBFS was performed for four different sources to axis of gantry rotation distances (SAD) based on:

A_eff_ (Ci) = RDR (dmax, FBFS) / (f_w_ × A_eq_ × BSF(FBFS) × ISL × Ãx ROF(FBFS) xSUF

With:

f_w_ exposure to dose in water (‘Roentgen to rad’) correction factor. f_w_ (Co-60) = 0.971[81]

A^eq^ transmission factor. A^eq^ (Co-60) = 0.99[81]

BSF back scatter factor. BSF (4×37.5 cm^2^) = 1.025 (interpolated from[82])

ISL inverse square law correction. ISL = (100 cm/SAD)^2^

Γ gamma factor. Γ(Co-60) = 1.297 R/(hr Ci)[82]

ROF relative output factor for reference field size to maximum field size (‘head scatter factor’). ROF (4×37.5 cm^2^) = 0.93

SUF source usage factor including self-attenuation of the source. SUF = 0.85 for a single source.

The activity required for the sources can also be calculated from the desired treatment time, T, the cranio-caudal dimension of a typical target, L and the anticipated intensity modulation required for the delivery. In a typical example, consider a 10 cm long target that needs to be covered by two adjacent 2 cm thick fan beams. One possible scenario would be a couch movement of 2 cm per minute with a rotation period of one rotation per minute. In this case, the pitch factor, p, defined as couch movement per rotation in units of fan beam thickness, would be 0.5, a typical value for helical tomotherapy planning.[83,84] In helical tomotherapy the pitch factor is in general smaller than 1 to ensure adjacent helices are overlapping, thereby reducing any problems with beam junctioning. The wide penumbra in Cobalt beams, the availability of two adjacent fan-beam rows and two opposing sources reduces the junctioning problems further.

Including a ramp up and ramp down of dose at the superior and inferior end of the target,[56] one would require about nine rotations for the target length coverage of 10 cm resulting in a treatment time of 9 min. In this scenario, any voxel of the target would be exposed to each source for 2 min each. As the radiation delivery is intensity modulated, not all MLC leaves, which allow primary beam to reach the target, are open for the total delivery period. In the present calculation a relative opening factor of 0.5 is employed which indicates that each leaf ‘seeing’ the target is open only for approximately half of the time possible. If one assumes in addition that the average attenuation within the patient reduces the incident fluence by 1/3 at the target, one requires an incident dose without attenuation at centre of rotation of approximately 1.5 Gy per source each to deliver a dose of 2 Gy per fraction to the target. The same calculation would apply for smaller pitches and proportionally faster rotation periods.

The average dose rate at depth of maximum dose of a Co-60 source of 12.8kCi activity was found by Glasgow to be 205 cGy/min in a 10×10 cm^2^ field at 1 m distant from the source in a commercial Co-60 unit.[76] At the 75 cm distance of the MRI Co tomotherapy unit this would yield a dose rate of 3.5 Gy assuming the fan beam of 4×37.5 cm^2^ produces somewhat less scatter than the 10×10 cm^2^ reference field. Following this, each of the two Co-60 sources requires an activity of around 220 TBq (6000 Ci), which is in good agreement with the results of the calculations shown in Figure 6. MDS Nordion, one of the major manufacturers of Co-60, offers sources with 1.5 cm diameter up to an activity of 8900Ci (personal communication P D'Amico, MDS Nordion).

Cobalt itself is a ferromagnetic metal. Therefore, it is proposed to use a low magnetic compound such as Cobalt sulfide (CoS) for the Cobalt source to minimise the effect of the magnetic field on the source. CoS has a molar magnetic susceptibility of 22.5×10^−5^ cm^3^/mole which is one of the lowest of the cobalt alloys. CoS (‘sycoporite’) has a melting point in excess of 1000°C and a physical density of 5.45 g/cm^3^ with 65% of the weight being Cobalt. Assuming a specific activity of Co-60 of 300 Ci/g[76,85] each of the sources must contain approximately 20 g of Co-60 that would fill a volume of 6 cm^3^ in the case of CoS. With a source diameter of 1.75 cm this requires a source of less than 2.5 cm thickness which is a common source dimension.[76]

**Figure 7:**
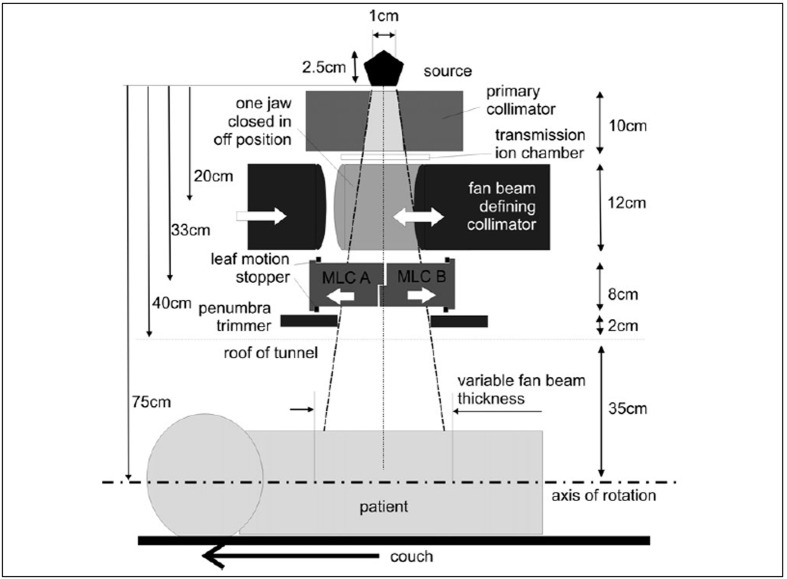
Side view of the proposed unit with dimensions

There is scope to optimise the source configuration for the delivery of fan beams. In the case of CoS the physical density of the material is more than 50% lower than pure cobalt metal. This would provide scope for increasing the source height to diameter ratio without significantly affecting self absorption compared to pure cobalt sources, thereby improving the geometry of the source. As the radiation will be delivered in a helical fashion, the beam penumbra in superior/inferior direction does not need to be very sharp. Nevertheless, due to the fact that the fan beam defining collimator is relatively close to the source, the current system design includes a penumbra trimmer as shown in Figures 5 and 7 to improve the penumbra in superior/inferior direction. This allows scope to reduce the source dimension within the plane of gantry rotation while increasing its size in direction orthogonal to the plane (i.e. patient's superior/inferior direction).

Compared to a linear accelerator, the dose distribution in a Cobalt beam is more homogenous and flattening filters are not commonly used. Also, the commercial helical tomotherapy unit does not use a flattening filter[77] as the intensity modulation can ‘flatten’ any beam profile if required. This arrangement favours Cobalt-60 as a radiation source as it effectively increases the output in all parts of the beam off central axis.

A less scientific but nevertheless important concern is that radioactive sources are often regarded as ‘out of fashion’ for teletherapy. Disposal and source changes pose problems for radiation safety. However, for the present proposal a radioactive source is ideally suited as it provides stable output without power requirements. This advantage is also realised in other advanced radiotherapy delivery systems such as high dose rate brachytherapy, the Gammaknife unit for stereotactic radiotherapy procedures,[86] a novel design for a Cobalt unit with multiple sources[87] and the RayView device mentioned in the introduction (www.rayview.com).

More topically, investigations in Kingston, Canada over the last 5 years have indicated that the concept of tomotherapy dose delivery is well suited to Cobalt-60 (^60^Co) sources.[88–90] This work was initially undertaken to investigate the usefulness of Co-60, a source with a 50 year long history that helped establish the foundation for high-energy radiation therapy, as a radiation source in modern conformal radiation therapy. The work supports the contention that while Co-60 has steadily fallen out of favour in clinical practice over the last two decades, this has not been because of the properties of the radiation beam, but rather because Co-60 units have not kept pace with modern progress in treatment technology.[91]

### Features of the on-board MRI system

The primary objective of the MR scanner is to acquire images with high spatial fidelity for verification of patient and organ location. Recent experience with open MR systems and with MRI radiation therapy simulators[47,48,65] suggest that this is readily achievable with a MR system with a relatively low field strength around 0.2T. The present system is based on a field strength of 0.25 T. The low field strength would be advantageous as it reduces image distortion due to an object (i.e. the patient) in the system.[92] The requirements for field homogeneity and gradient linearity remain high.[93] However, any imperfections of these components can be determined in phantom studies and corrected for prospectively.[94] An additional advantage of the low field system is that the fringe field in the rotating gantry is also relatively low, which reduces safety hazards.

Hayashi *et al.* have discussed the advantages of low field MR scanners in 2004.[95] According to this work, low field scanner image quality is continuously improving as they are benefiting from improved image acquisition and handling software. They have particular strength in terms of flexibility, patient safety and cost effectiveness, the very features of interest in the present design.

**Figure 8:**
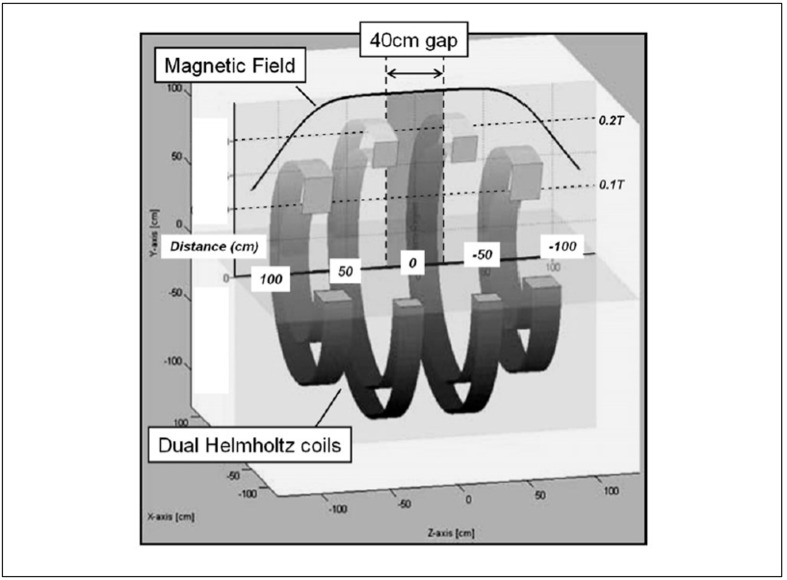
Dual Helmholtz coil design proposed for the CoMRI tomotherapy unit. The field strength is plotted along the central axis based on a maximum field of 0.25T

The magnet design based on Helmholtz coils has been employed previously in a commercial open MR system marketed by General Electric (GE) several years ago as ‘GE Horizon.’ This GE 0.5T open magnet system was specifically designed for interventional and intra-operative imaging where the space between the coils could be used for access to the patient. In the context of MiCoTo, the Helmholtz design has the advantage of producing a relatively strong homogenous magnetic field while leaving a gap for the tomotherapy gantry ring. The support for the gantry comes from two frames that also separate the rotating gantry from the two MRI segments housing the magnetic field coils. It is proposed to utilise a dual Helmholtz coil design as originally discussed by Garrett[96] and further developed by Franzen[97] and later Kaminishi.[98] Using this design as illustrated in Figure 8, it can be shown that two sets of coils with a bore diameter of approximately 1 m and a gap of 40 cm can produce a 40 cm long zone with a uniform field with less than 15 ppm variation.[99] The resulting gap is wide enough for a ring gantry accommodating two Co-60 sources and the associated beam delivery system as discussed above.

The present MR integrated Cobalt tomotherapy unit is designed primarily with pelvic radiotherapy treatments in mind. This applies not only for prostate cancer, where IMRT is already widely used.[100,101] but also for gynaecological malignancies where concurrent chemotherapy (bone marrow sparing desirable) and potentially involved lymph nodes create a need for better controlled dose delivery.[102,103] In this case, the superior soft tissue contrast of MRI will be beneficial for target localisation and the relatively large size of the beam penumbra is compatible with typical intrafraction motion of targets during radiotherapy.[104] It is anticipated that the primary function of the MR imaging system for pelvic radiotherapy will be conventional T1 and T2 weighted imaging. Therefore, imaging sequences, which are available on most commercial systems, can be employed.

It is an advantage that the Co-60 beam does not require beam steering. Therefore, significant components of the electronics typically required for linear accelerators are not necessary reducing the potential problems with computers operating on a rotational gantry in a strong magnetic field. However, other computer systems associated with the unit would still have to operate in the magnetic field. This includes the MLC control computer and all beam-monitoring devices which require at least some gantry mounted computing equipment to ensure fast interlocking and control without the need to transmit signals via the rotating gantry slip-ring. If a one of the Co-60 sources is used for CT scanning as discussed above, the detector system must also operate in a strong magnetic field.

### Treatment planning

Because of the complexity of the delivery patterns, treatment planning for helical tomotherapy can only be performed using inverse treatment planning.[105–109] It is proposed to use a delivery technique with a limited number of projections per rotation similar to the commercial helical tomotherapy unit HiArtR (TomoTherapy Inc - http://www.tomotherapy.com/, compare also).[83,84] In this system 51 projections per rotation are used and it would not be necessary to develop a completely new treatment planning system as the same treatment planning system can be used after an appropriate Co-60 beam has been commissioned for the superposition/convolution dose calculation engine. It is therefore anticipated that an existing treatment planning system can be utilised for the planning process.

Modifications in the treatment planning system would be required to take into account the different geometry of the system, the different design of the MLC and the potential inclusion of magnetic field effects on the dose spread array used in the superposition/convolution dose calculation.[110–113] Additional modification would be required for the verification and quality assurance tools.[114]

It needs to be also considered that the dose distribution in the patient will be affected by the presence of a strong magnetic field. While the primary photons are independent of the magnetic field, the path of the secondary electrons will be influenced by the field. Raaymakers *et al.*[111] have shown that in a 6MV accelerator produced X-ray beam this effect increases the penumbra by 1mm. In addition to this, the build-up region is reduced. Both effects are small in a rotational delivery using Co-60 compared to megavoltage linear accelerator X-rays.

An additional advantage of quasi-monoenergetic cobalt radiation is that the spectrum of primary photons is not affected by beam hardening and thus independent of the location within the patient. Therefore, dose calculation algorithms such as superposition convolution[110] can take a modification of the deposition kernel due to the static magnetic field explicitly into account.

## Possible future developments

### Higher spatial resolution in delivery

In the case of head and neck tumours, it may be necessary to increase the spatial resolution of the dose delivery in future MR integrated Cobalt Tomotherapy (‘MiCoTo’) systems. This can be achieved in several ways that need to be employed together for best effects. Firstly, different MLC leaf configurations with smaller leaf widths and more leaves would be possible. This could include variable leaf sizes with smaller leaf spacing only in the centre (e.g. 15×1 cm + 12×2 cm leaves). Secondly, the fan beam thickness could be reduced; however, the significant drop in output due to partial source occlusion must be considered.[115] Also, the source size could be reduced and redesigned. This would likely result in reduced source strength and one needs to accept either increased treatment times or less intensity modulation. Alternatively, it is possible to increase the number of sources (and MLCs) around the ring gantry. Given the fact that Cobalt sources do not require extensive electronics there would be adequate space on the ring gantry to accommodate several additional sources.

### On-line imaging and imaging of moving objects

It will be a major benefit in the development of image guided and adaptive radiotherapy if the imaging could occur during treatment. This ability will be one of the major advantages of the proposed unit as interference between imaging and treatment is unlikely because two independent physical principles are used. The real time aspect of the image acquisition during treatment can be useful for beam gating[116,117] and/or control of motion adaptation of the radiation delivery.[6,23] In this case it can be assumed that image information from a limited number of planes[104] will suffice.

If imaging is performed during treatment, couch movement must be taken into consideration. This couch movement will typically be slow and not exceed 5 cm per minute. As such, the couch motion during the repetition time TR in the imaging sequence would be typically less than 1 mm. However, it will be necessary to account for the slice location in the patient in superior/inferior direction as this will depend on both, gradient strength and pulse frequency as well as couch position. Even more complex reconstruction methods must be developed if sagittal and coronal slices are to be acquired. The image reconstruction software must take these additional parameter into consideration. A MRI visible marker at the side of the treatment couch could be useful to unambiguously identify the location of the couch in the images.

### The need for high field MiCoTo

The MR integrated Cobalt tomotherapy unit described here utilizes a non-superconducting electromagnet. This allows for simple, cost effective, design without cryogenic requirements and the magnetic field can be relatively quickly turned off which would allow for ‘field free’ treatment after the imaging process. It has also been shown that treatment planning can be performed from low field MR images[118] allowing potentially for dose reconstruction.[30,34] However, there will always be concerns regarding the inferiority of low field systems compared to high field MRI.[119] Therefore, a high field MiCoTo unit could be established, if the concept based on relatively low field strength proves to be feasible and clinically useful.

This would require the development of a system based on a superconducting magnet. While a considerable challenge this development might enable the extension of the MR guidance to functional imaging and/or spectroscopy for assessment of daily treatment progress.[120] This feature combined with the better spatial resolution afforded by a high field system would be of great interest, for example, in the treatment of head and neck cancers. As more tools for MRI acquisition become available for oncological imaging one may be able to perform on-line MR spectroscopy while radiation treatment is performed.

### Alternative imaging modality using Cobalt-60 CT

As the ring gantry needs to be balanced the two therapy sources are located on opposite sides of the gantry. It would be possible to add another radioactive source (or a diagnostic x-ray tube) 90degrees offset on the gantry. If a CT detector array were mounted on the opposite side, it would be possible to also reconstruct CT scans and attenuation maps that can compliment the MRI information and be used for tissue densitometry and dose calculations.[90]

### MRI gel dosimetry

In all advanced radiotherapy delivery techniques quality assurance is of utmost important. Unlike conventional radiotherapy, the verification of treatment plans for individual patients undergoing IMRT requires an absolute dose measurement and the assessment of three dimensional dose distributions. For the latter, radiographic film is typically used in multiple planes, but many researchers have proposed radiation sensitive gels for this purpose.[121–123] The present unit would be ideally suited for this as MRI can be used to evaluate the gels after or even during the radiation delivery. This would overcome problems with diffusion in Fricke type gels[124] and would allow the use of these easy-to-manufacture gels in treatment verification.

## Conclusion

A novel concept for adaptive radiotherapy, an MR integrated Cobalt Tomotherapy (MiCoTo) system has been developed. At this stage this is only a proposal which will require experimental realisation. The aim of the present paper was to review existing methods for image guidance in radiotherapy. Based on the need to improve soft tissue contrast for image guidance during delivery, the concept of an MR integrated Cobalt Tomotherapy unit was presented and the crucial components for such a development identified.

The unit combines the excellent imaging capability of magnetic resonance imaging with the powerful radiation delivery approach of helical tomotherapy. A Cobalt source is employed as radiation source to provide a stable, reliable output during gantry rotation. The initial proposal includes two radioactive sources on opposite sides of a rotating ring gantry to increase the overall radiation output. An additional reduction in treatment time can be achieved using a dual row binary multi-leaf collimator. The proposed unit is designed for pelvic and abdominal radiotherapy, however, modifications of the design could also result in units suitable for the treatment of targets in other parts of the body.

The practicality of the proposal needs to be proven over the next few years by realising all steps identified above and building a working prototype unit. This will require significant resources but appears likely to be feasible. In any case, it appears to be important to further develop the concept of IGRT by including MRI. The advantages of the proposed Cobalt MRI tomotherapy unit are significant and include the following:

- Two entirely independent physical concepts are used for imaging and treatment
- Great potential for target localisation
- Simple radiation unit design
- Stable output
- Imaging is non-invasive and does not use ionising radiation - therefore multiple images can be taken even on a single day
- Significant research potential for on-line monitoring of targets and normal structures during or directly after irradiation
- Truly 3D datasets including sagittal and coronal slices can be acquired
- Patient QA can be performed with radiation sensitive gels. They can be irradiated and evaluated in situ using MRI.

The system delivers highly conformal radiation dose distributions using Co 60 sources and pneumatically driven MLCs. Both are relatively easy to maintain and do not rely on expensive power and air conditioning systems. Verification can be performed with cheap and easy to manufacture Fricke dosimetric gels. As such, the proposed system may also be of interest for developing countries as it allows the introduction of state of the art radiotherapy with relatively low maintenance costs and without the need for extensive infrastructure.
